# Phylogenetic and *In Silico* Functional Analyses of Thermostable-Direct Hemolysin and *tdh*-Related Encoding Genes in *Vibrio parahaemolyticus* and Other Gram-Negative Bacteria

**DOI:** 10.1155/2014/576528

**Published:** 2014-07-08

**Authors:** Sushanta K. Bhowmik, Gururaja P. Pazhani, Thandavarayan Ramamurthy

**Affiliations:** National Institute of Cholera and Enteric Diseases, P-33, CIT Road, Scheme XM, Beliaghata, Kolkata 700010, India

## Abstract

Emergence and spread of pandemic strains of* Vibrio parahaemolyticus* have drawn attention to make detailed study on their genomes. The pathogenicity of* V. parahaemolyticus* has been associated with thermostable-direct hemolysin (TDH) and/or TDH-related hemolysin (TRH). The present study evaluated characteristics of* tdh* and* trh* genes, considering the phylogenetic and* in silico *functional features of* V. parahaemolyticus* and other bacteria. Fifty-two* tdh* and* trh *genes submitted to the GenBank were analyzed for sequence similarity. The promoter sequences of these genes were also analyzed from transcription start point to −35 regions and correlated with amino acid substitution within the coding regions. The phylogenetic analysis revealed that* tdh* and* trh *are highly distinct and also differ within the* V. parahaemolyticus* strains that were isolated from different geographical regions. Promoter sequence analysis revealed nucleotide substitutions and deletions at −18 and −19 positions among the pandemic, prepandemic, and nonpandemic* tdh* sequences. Many amino acid substitutions were also found within the signal peptide and also in the matured protein region of several TDH proteins as compared to TDH-S protein of pandemic* V. parahaemolyticus*. Experimental evidences are needed to recognize the importance of substitutions and deletions in the* tdh* and* trh *genes.

## 1. Introduction 

*Vibrio parahaemolyticus* is a Gram-negative bacterium, which is a part of the normal flora of marine and estuarine waters. Despite its halophilic nature, this pathogen has also been isolated from fresh water and freshwater fishes. Genetically and by serology,* V. parahaemolyticus* strains are very diverse. During February 1995, an unusual incidence of* V. parahaemolyticus* belonging to serovar O3:K6 was recorded among acute diarrheal cases in the Infectious Diseases Hospital, Kolkata [1]. Since 1996, this O3:K6 serovar has been associated with several outbreaks in different countries and hence designated as the pandemic strain [1]. The O3:K6 and its genetically related serovars of* V. parahaemolyticus* are now documented as a pandemic clonal complex and have been related to its global spread [1].

The pandemic serovars of* V. parahaemolyticus* are now considered as an emerging pathogen in Asia and coastal regions of the United States [2] due to several episodes of large seafood-associated infections. This pathogen has been frequently detected in shellfish than in sediment or water samples [3]. Apart from gastroenteritis, wound infections and septicemia are the other major clinical manifestations caused by pathogenic strains of* V. parahaemolyticus*. This* Vibrio* causes infections in human due to consumption of raw or undercooked seafood or the wounds exposed to warm seawater. Patients with chronic liver diseases and leukemia are predisposed to septicemia caused by* V. parahaemolyticus*, which is sometimes fatal [4]. Gastroenteritis is caused by diverse serovars of* V. parahaemolyticus*; however, strains of O3:K6 with unique* toxRS* gene sequence are distributed throughout the world as a pandemic serovar. The O3:K6 serovars that lacked the* toxRS *sequence isolated before 1996 are known as prepandemic strains of* V. parahaemolyticus*. Serovar O3:K6 continued to exist in the environment, confronting several ecological and immunological changes in the population resulting in progression of several other new pandemic serovars.

Enterotoxicity of this pathogen is attributed to the extracellular production of a putative virulence factor, the thermostable-direct hemolysin (TDH). TDH has been phenotypically shown as the *β*-type hemolysin on Wagatsuma agar, which is also known as the Kanagawa phenomenon (KP). Apart from the KP-test, the purified TDH has been tested in myocardial cells [5], rabbit ileal loops [6], and enzyme-linked immunosorbent assay. The purified TDH caused a dose-dependent increase in intracellular free calcium in both Caco-2 and IEC-6 as detected by microspectrofluorimetry [7]. Significant lethal activity of TDH was also demonstrated in the murine infection model [8]. Sometimes, the KP-negative strains of* V. parahaemolyticus *produce a TDH-related hemolysin (TRH). The TRH has similar physicochemical properties like TDH, but it is liable at temperature 60°C [9]. The pathogenic strains of* V. parahaemolyticus* that harbor only the* tdh *and express KP were found to be associated with acute diarrheal infection and epidemics [10]. The environmental strains that cause extraintestinal infections may differ in this virulence profile [11]. Generally, the detection rate of* trh* in clinical strains is very less but comparatively more in environmental strains. However, high frequencies of* tdh* and* trh* genes positive strains have been detected recently in a pristine estuary of US [12]. Considering their importance, detection of these virulence marker genes is important to differentiate pathogenic strains from nonpathogenic* V. parahaemolyticus*.

TDH is associated with type-three secretion systems (T3SSs) [13, 14].* V. parahaemolyticus* has two sets of T3SS genes on chromosomes 1 and 2 (T3SS1 and T3SS2, resp.). The T3SS1 can induce cytotoxicity [14], whereas the T3SS2 can induce cytotoxicity in Caco-2 cells and also plays an important role in fluid secretion in the ileal loops [15]. Comparative genomic analysis confirmed that the T3SS2-containing PAI was conserved in KP-positive strains [16].

*V. parahaemolyticus* that lacks typical* tdh* and* trh* may phenotypically express hemolytic activity due to the presence of its variant forms. These variants have considerable homology with established prototypes of* tdh/trh*. In this study, we assessed molecular diversity of* tdh* and* trh* gene sequences in order to understand the phylogenetic relationship and* in silico* functionality among* V. parahaemolyticus* and other Gram-negative strains reported from different geographical areas. In* V. parahaemolyticus,* five* tdh* alleles have been identified, namely,* tdh1* to* tdh5*, with similar biological activities [17]. These alleles have >96.7% sequence identity. However, expression of these alleles varied due to the defect in their promoter activities [18].

## 2. Materials and Methods

A total of 5 diverse bacteria with fully sequenced hemolysin genes (*tdh*,* trh*, and other hemolysin genes of* V. parahaemolyticus*) were selected and aligned for phylogenetic analyses (maximum parsimony and neighbor-joining methods) using MEGA software version 5.2 [19]. Nucleotide sequence length of 570 bp and alignment score of 13 were sustained to include majority of hemolysin encoding genes and aligned accurately from diverse bacterial strains. Considering these criteria, hemolysin genes represented by 52 strains, including 47* V. parahaemolyticus* (37* tdh*, 8* trh,* and 2 of hemolysin III and a delta* tdh* genes), 2* V. cholerae* (one of each of* V. cholerae* non-O1, non-O139 (NAG), and serotype O1), and one of each of* V. mimicus* (*tdh*),* Vibrio hollisae* (*tdh*), and* Listonella anguillarum* (*trh*), were included in this analysis. A phylogenetic tree was constructed by bootstrap analysis through 1000 replicates. In addition to phylogenetic analysis, promoter regions of* tdh* genes harboring* Vibrio *spp. and their amino acids were analyzed.

## 3. Results and Discussion

**Figure 1:**
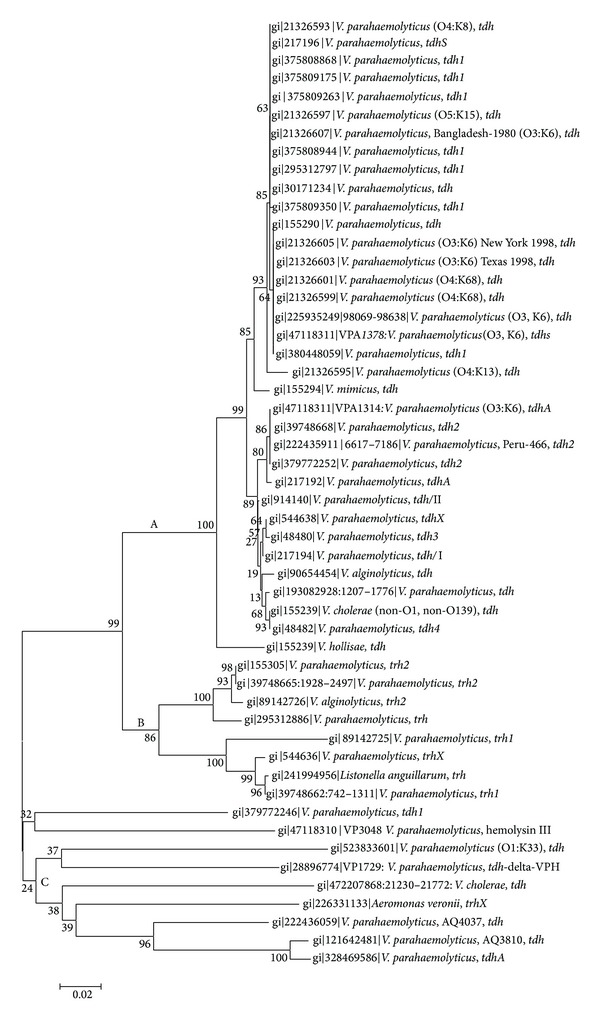
Neighbor-joining phylogenetic tree obtained by the analysis of* tdh* and* trh* genes. Bootstrap values are presented next to the tree nodes. The branch of the tree is not proportional to evolutionary distance. The bar represents 0.02 nucleotide substitution per site.

Hemolysin is a potential virulence factor in many bacterial pathogens. It is well known that the TDH has a combination of biological actions including hemolysin, cardiotoxicity, and enterotoxicity. The severity of diarrheal illness caused by this bacterium is closely related to the presence of two types of* tdh* and* tdh*-related genes [20]. Depending on the environmental conditions, these virulence genes also play an important role in the stress tolerance in* V. parahaemolyticus* [21]. The results of phylogenetic analysis of* tdh* and* trh* genes are shown in Figure 1. In the phylogenetic tree, three distinct clades (A to C) were identified. In clade A,* tdh* gene from diverse serogroup of* Vibrio* spp. had 85 to 100% sequence similarity within the coding region. Clade A contained more of* V. parahaemolyticus* nonpandemic strains (91%) than pandemic strains (8%). Clade B had the* trh* sequences of* V. parahaemolyticus* and* Listonella anguillarum*. Clade C contained mostly the nonpandemic strains of* V. parahaemolyticus*.

So far, five* tdh* genes have been identified in plasmids and chromosomes of* Vibrio* spp. [22] and their sequence displayed >96.7% identity with similar biological activity [18]. These* tdh* genes not only are restricted to* V. parahaemolyticus *alone but also have been documented in other* Vibrio* species such as* V. hollisae*,* V. mimicus*, and* V. cholerae* [22]. Typical hemolysin-producing* V. parahaemolyticus* strains carry two copies of* tdh* genes (*tdh1* and* tdh2*) in their chromosomes [22]. Strains that harbor any one of these genes have been associated with weak or negative hemolytic activity. The gene* tdh2* holds 97.2% homology with* tdh1* and was found primarily responsible for the phenotypic expression of hemolytic activity [22]. These two genes are designated as* tdhA* and* tdhS* [23] and detected in a gene cluster known as* tdh* pathogenicity island (*tdh-*PAIs) of pandemic serovars [24]. These* tdh-*PAIs are very similar in many epidemic strains of* V. parahaemolyticus* but are absent in a prepandemic strain AQ4037 [24]. Although this PAI has been detected in another prepandemic strain of AQ3810, the* tdhS* gene orientation was reversed [24]. The difference in the presence of* tdh-*PAIs in the pandemic strains and positioning of* tdh* genes among prepandemic strains indicated that these genes have been acquired by lateral gene transfer in* V. parahaemolyticus*. This hypothesis was supported by differences in the G + C content of the* tdh*-PAI and the rest of the genome [25].

In the phylogenetic analysis, the pandemic and prepandemic strains were placed in A and C clades. The size of the typical* tdh* coding sequence was 570 bp. However, in this analysis, we have included only the published full length sequences. The* trh *gene from* Aeromonas veronii* biovar Veronii sequences has also been analyzed for this study. Since all the three* trh *sequences are identical, we have considered one to examine its relation to the* trh *of* V. parahaemolyticus*. The* trh* sequences of* Aeromonas* spp. are highly diverse and their bootstrap values remained less than 50%. Clades A and C are the two clusters in which diverse hemolysin encoding genes have been grouped. Clade A contained* tdh* of pandemic and nonpandemic strains. The* tdh *sequence of pandemic serovars exhibited 86–99% bootstrap homology with nonpandemic serovars and* trh* gene of the* V. parahaemolyticus* ATCC strain 17802 (serovar O1:K1) [26]. In addition, the* tdh* also showed 86% homology with* trh* of* Listonella anguillarum*, which is a member in the family Vibrionaceae. In clade A,* tdhA* of RIMD2210633 had 86% sequence homology with a Peruvian pandemic strain Peru-466 and the* tdhS* had 64% homology with* tdh1* of Indian pandemic strain K5030 [24]. This genetic comparison demonstrates that pandemic strains isolated from several geographical areas displayed sequence dissimilarity within the* tdh* coding region. However, clade A contained* tdh* genes of* V. parahaemolyticus* from US, Bangladesh, and Russia. The pandemic serovars from US and Bangladesh had 93% sequence homology [27] but the information on the types of Russian serovars is not available.

In this study,* tdh* of* V. cholerae* non-O1 and non-O139,* V. mimicus,* and* V. hollisae* showed sequence homology with* tdh* of* V. parahaemolyticus*. However, the bootstrap similarities are distinct (Figure 1). Although these organisms had some sequence similarities within the coding regions of hemolysin encoding genes, a comparative analysis showed that they had different flanking regions as compared to* V. parahaemolyticus* [22]. Honda et al. [28] reported the presence of plasmid-encoded TDH in some of the environmental strains of* V. cholerae* non-O1 and non-O139 (also called nonagglutinable (NAG) vibrio) strains. Type-III secretion system (T3SS) located in ~49.7 kb genomic island has been identified in NAG strains, which has a strong homology with T3SS2 of* V. parahaemolyticus*. The TDH and TRH encoding genes in NAG strains have been identified either within [29] or outside [30] the T3SS genomic island. Although* V. hollisae* strains had T3SS2 island, TDH/TRH was not reported as a part of this island [31].

**Figure 2:**
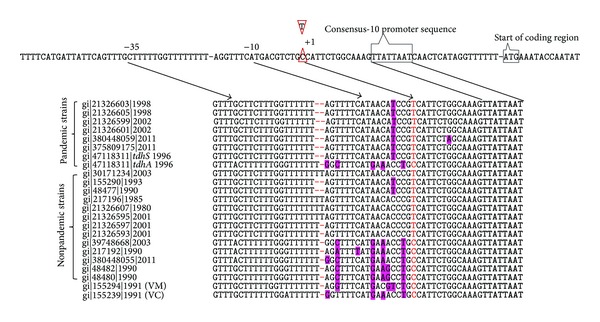
Comparison of promoter nucleotide sequences of* tdh* genes of* V. parahaemolyticus*,* V. mimicus,* and* V. cholerae*. VM,* V. mimicus*; VC,* V. cholerae* non-O1 and non-O139.

It has already been established that the expression of* tdh* and* trh* genesis different due to defect in the promoter regions [18, 27]. In* V. parahaemolyticus*, changes in the promoter sequences of different* tdh* genes have shown considerable variation in the expression of KP [18]. It was shown that the nucleotide sequence positions from −35 to −10 of* tdh* gene promoter act as a hotspot and nucleotide substitution at −34 from A to G affects the expression of hemolytic activity [18]. This −34 position corresponds to −35 in our realigned sequence comparison (Figure 2). In a recent finding, it was revealed that, in the absence of any substitution or an additional mutation at position −3 (substitution of G to A) relative to −10, sequence of promoter region could change the expression of hemolysin [32]. This information facilitated analyzing the nucleotide sequences of promoter regions from transcription start point to −35 position of* tdhS* of RIMD2210633 with other available 23 promoter sequences of* tdh* from the GenBank (except* tdh5*, which was not available in the database). The gene* tdhS* is highly transcribable under the influence of the promoter region, which was associated with stronger KP [18]. However, in the comparative analysis, instead of substitution at −3 position, we have detected nucleotide changes at −2 (C for T), −4 (T for C), −5 (A for G), −6 (C for A), −8 (A for G), −15 (T for C/A/G), and −17 (A for G) in* tdhS* of RIMD2210633, which is a pandemic serovar O3:K6. Site-directed mutagenesis experiments are required to address the importance of these substitutions. In addition to nucleotides, positions −18 and −19 relative to the −10 were found to be altered among nine* tdh* genes, which are intact, mostly in pandemic serovars such as O3:K6, O4:K68, and O1:KUT (K antigen untypable) (Figure 2). However, these changes were absent in four* tdh* genes sequenced from strains of ATCC 17803 (gi|30171234), T4750 (gi|217196), and Bangladesh-1980 (serovar O3:K6) (gi|21326607) and in sequence gi|21326595 (from serovar O4:K13) (Figure 2). Among the 10* tdh* genes, a nucleotide deletion at position −18 was found among pandemic, prepandemic, and one of each of* V. cholerae* non-O1 and non-O139 and* V. mimicus* strains. We also analyzed protein sequences in the promoter region of all the strains. The TDH consists of 189 amino acids, of which first 24 amino acid residues belonged to signal peptide. A site-directed mutagenesis study on the remaining 165 amino acids residues has shown that Trp^65^  and Leu^66^ are very important in the hemolytic activity of TDH and any change in these residues could reduce its activity [33]. In addition to these residues, Arg^46^, Gly^62^, Thr^67^, Gly^86^, Glu^116^, and Glu^138^ were also shown to be vital for the hemolysis [33, 34]. TDH has one intramolecular disulphide bond between Cys^151^ in *β*10 and Cys^161^ in the 310 helix [35]. This contiguous positioning of Cys^151^ and Cys^161^ suggests the formation of side channels and influences the hemolytic activity of TDH. These two Cys residues were also found to be highly conserved in all the TDH. However, mutations in other positions were detected when comparing TDH sequences of RIMD2210633 with others (Table 1). TDH-A of RIMD2210633, TDH3, TDH4,* V. mimicus* TDH,* V. cholerae* non-O1 and non-O139-TDH, and other prepandemic strains of* V. parahaemolyticus* had amino acid substitutions within the signal peptide at positions 3 (tyrosine for histidine), 4 (glutamine for arginine), and 23 (phenylalanine for serine) as compared to RIMD2210633. Interestingly, these groups of TDH amino acids do not have histidine in the signal peptide, which is essential in the protein active or binding sites. In a* V. mimicus* (VmTDH), substitution at position 4 was absent. Except in one, all* tdh* sequences that contained double deletion in the promoter sequence at −18 and −19 gained Gly^99^ in the place of aspartic acid (Asp^99^). The significance of this mutation needs to be evaluated.

It has been reported that* trh* gene has two alleles, namely,* trh1* and* trh2*. The sequences of* trh1* and* trh2* share 84% and 68% similarity with* tdh2*, respectively [36]. In the initial studies, it was thought that downstream inverted repeat sequence (IRS) from −35 to −10 of* trh1* and* trh2 *may have some association with low expression of TRH [37, 38]. In the subsequent finding, it was reported that the promoter-bearing region was responsible for the low expression* trh *transcription rather than the role of IRS [36].

The* trh* harboring* V. parahaemolyticus* strains universally carries a urease gene (*ureR*);* V. parahaemolyticus* strains isolated from Asian countries always exhibit a strong correlation between the* ureR* gene and* trh* positivity [39]. However, the association between these two genes is not related in the transcription of* trh* [36]. In a clinical perspective, urease-positive phenotype is considered for elevated virulence in* V. parahaemolyticus* [40]. We did not find any differences in the promoter sequence between* trh* and* tdhS* of RIMD2210633, as reported before [36]. Recently, the whole-genome of* Oceanimonas* (strain GK1) belonging to the family Aeromonadaceae has been sequenced and a* tdh* gene has been detected in the chromosome [41]. The protein sequence of TDH matched with a TDH of* Aeromonas* spp., but not with the TDH of* V. parahaemolyticus*. Among the* Vibrio* species, only* V. alginolyticus* carried* tdh* and* trh* genes. The* trh* of gene of* V. alginolyticus* also shared considerable homology with* trh* of* V. parahaemolyticus* (data not shown).* trh* genes of* V. parahaemolyticus* and* Listonella anguillarum* have been placed in clade B (Figure 1).

Phylogenetic analysis suggested that there is a high level of sequence diversity in* tdh* and* trh* among* V. parahaemolyticus* strains and in other vibrios. Since these genes are carried by the transposon, they have been detected in many* Vibrio* spp. [42]. The reason for selective uptake of these genes only in* Vibrio* species needs to be investigated. Using this* in silico* approach, differences in promoter sequences were identified among the pandemic and nonpandemic strains of* V. parahaemolyticus*. Such differences are probably associated with differential transcription in* V. parahaemolyticus* strains. More experimental evidences may prove the importance of mutations detected in this study.

## Figures and Tables

**Table 1:** Comparison of the deduced amino acid sequences of the products of the *tdh* genes taken from GenBank.

Particulars of *tdh* genes	Positions of signal peptide			Positions of mature protein sequence															
	3	4	23	23	28	34	43	50	89	99	108	112	116	119	136	147	162	163	165
Pandemic strains																			
gi∣21326604∣1998 (O3:K6)	H	Q	S	T	Q	K	N	E	S	**G**	N	N	E	G	N	I	K	H	Q
gi∣21326606∣1998 (O3:K6)	H	Q	S	T	Q	K	N	E	S	**G**	N	N	E	G	N	I	K	H	Q
gi∣21326600∣2002 (O4:K68)	H	Q	S	T	Q	K	N	E	S	**G**	N	N	E	G	N	I	K	H	Q
gi∣21326602∣2002 (O1:KUT)	H	Q	S	T	Q	K	N	E	S	**G**	N	N	E	G	N	I	K	H	Q
gi∣380448060∣2011	H	Q	S	T	Q	K	N	E	S	**G**	N	N	E	G	N	I	K	H	Q
gi∣28901233∣RIMD, TDH S, 1996 (O3:K6)	H	Q	S	T	Q	K	N	E	S	**G**	N	N	E	G	N	I	K	H	Q
gi∣28901169∣RIMD, TDH A, 1996 (O3:K6)	**Y**	**R**	**F**	T	N	E	N	K	**H**	D	D	N	E	D	N	I	K	H	Q
Nonpandemic strains																			
gi∣48478∣1992	H	Q	S	T	Q	K	N	E	S	**G**	N	N	E	G	N	I	K	H	Q
gi∣155291∣1993	H	Q	S	T	Q	K	N	E	S	**G**	N	N	E	G	N	I	K	H	Q
gi∣21326608∣1980 (O3:K6)	H	Q	S	T	Q	K	N	E	S	D	N	N	E	G	N	I	K	H	Q
giI∣217197∣1985	H	Q	S	T	Q	K	N	E	S	D	N	N	E	G	N	I	K	H	Q
gi∣30171235∣ATCC 17803, 2003	H	Q	S	T	Q	K	N	E	S	D	N	N	E	G	N	I	K	H	Q
gi∣21326594∣2001 (O4:K8)	H	Q	S	T	Q	K	N	E	S	D	N	N	E	G	N	I	K	H	Q
gi∣375809176∣2011	H	Q	S	T	Q	K	N	E	S	D	N	N	E	G	N	I	K	H	Q
gi∣21326598∣ 2001 (O5:K15)	H	Q	S	T	Q	K	N	E	S	D	N	N	E	G	N	I	K	H	Q
gi∣21326596∣2001 (O4:K13)∗	H	Q	S	T	Q	K	N	E	S	D	N	N	E	G	N	**V**	—	—	—
gi∣155295∣Vm-TDH, 1991	**Y**	Q	**F**	T	**K**	K	**D**	E	S	D	N	N	E	D	N	**V**	**E**	H	**R**
gi∣39748669∣2003	**Y**	**R**	**F**	T	N	E	N	K	**H**	D	D	N	E	D	N	I	K	H	Q
gi∣217193∣1990	**Y**	**R**	**F**	T	N	E	N	K	**H**	D	D	**S**	E	D	N	I	K	H	Q
gi∣380448056∣2011	**Y**	**R**	**F**	T	N	E	N	K	**H**	D	D	N	E	D	N	I	K	H	Q
gi∣48483∣1990	**Y**	**R**	**F**	T	N	K	N	K	**N**	D	N	N	E	D	**D**	I	K	**Y**	**N**
gi∣155240∣VcNAG-TDH, 1991	**Y**	**R**	**F**	T	N	K	N	K	**N**	D	N	N	E	D	**D**	I	K	**Y**	**N**
gi∣48481∣1990	**Y**	**R**	**F**	**A**	N	K	N	K	**R**	D	N	N	**K**	D	N	I	E	H	**K**

*Truncated *tdh* sequence; Vm, *V. mimicus*; VcNAG, *V. cholerae* non-O1 and non-O139; Bang, Bangladesh; RIMD, RIMD2210633. Known serovars are mentioned in parentheses.
